# Supplementary figures and images for: Redirection of SARS-CoV-2 to phagocytes by intranasal sACE2-Fc as a universal decoy confers complete prophylactic protection
Source: eLife. 2026 May 18;14:RP108883. doi: 10.7554/eLife.108883 (PMC13183376; doi:10.7554/eLife.108883)

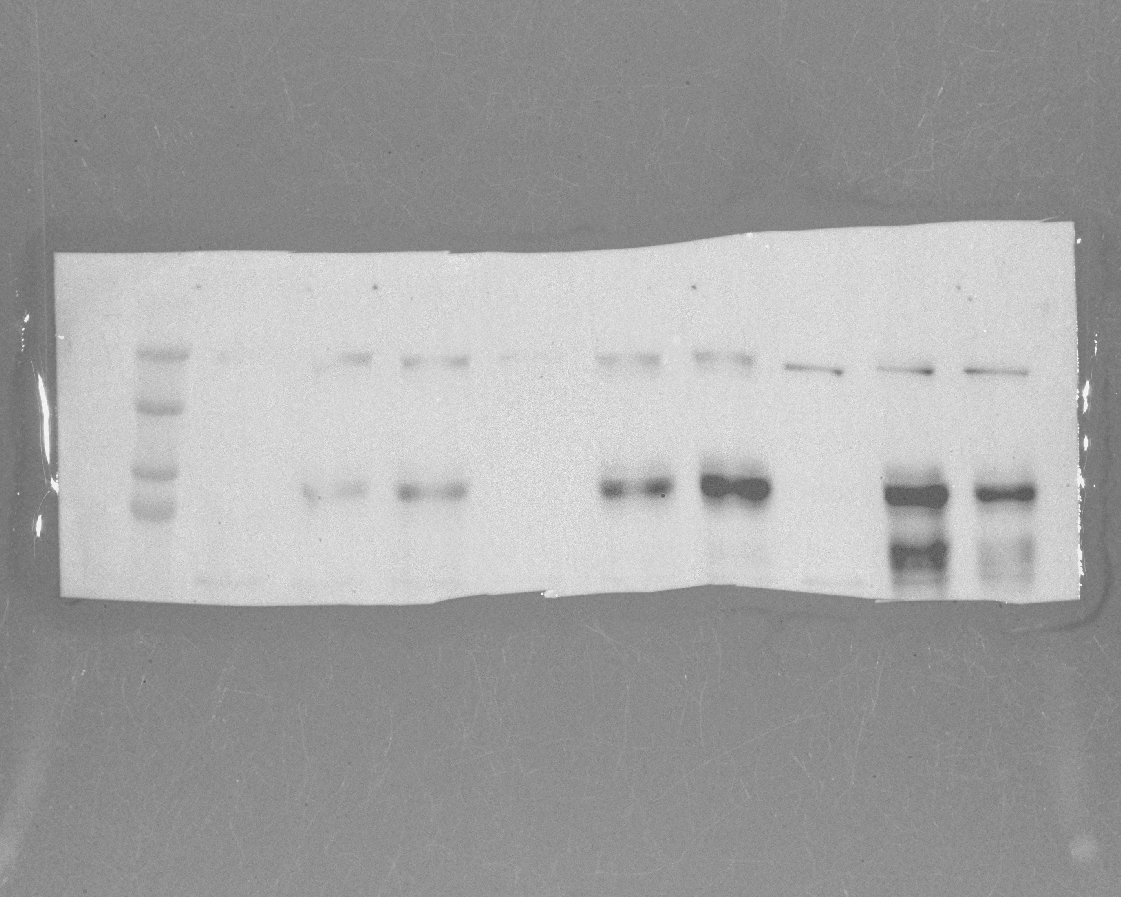

Supplement: Figure 6—source data 1. [file elife-108883-fig6-data1.zip › Figure 6-source data/Figure 6-source data 1.tif]

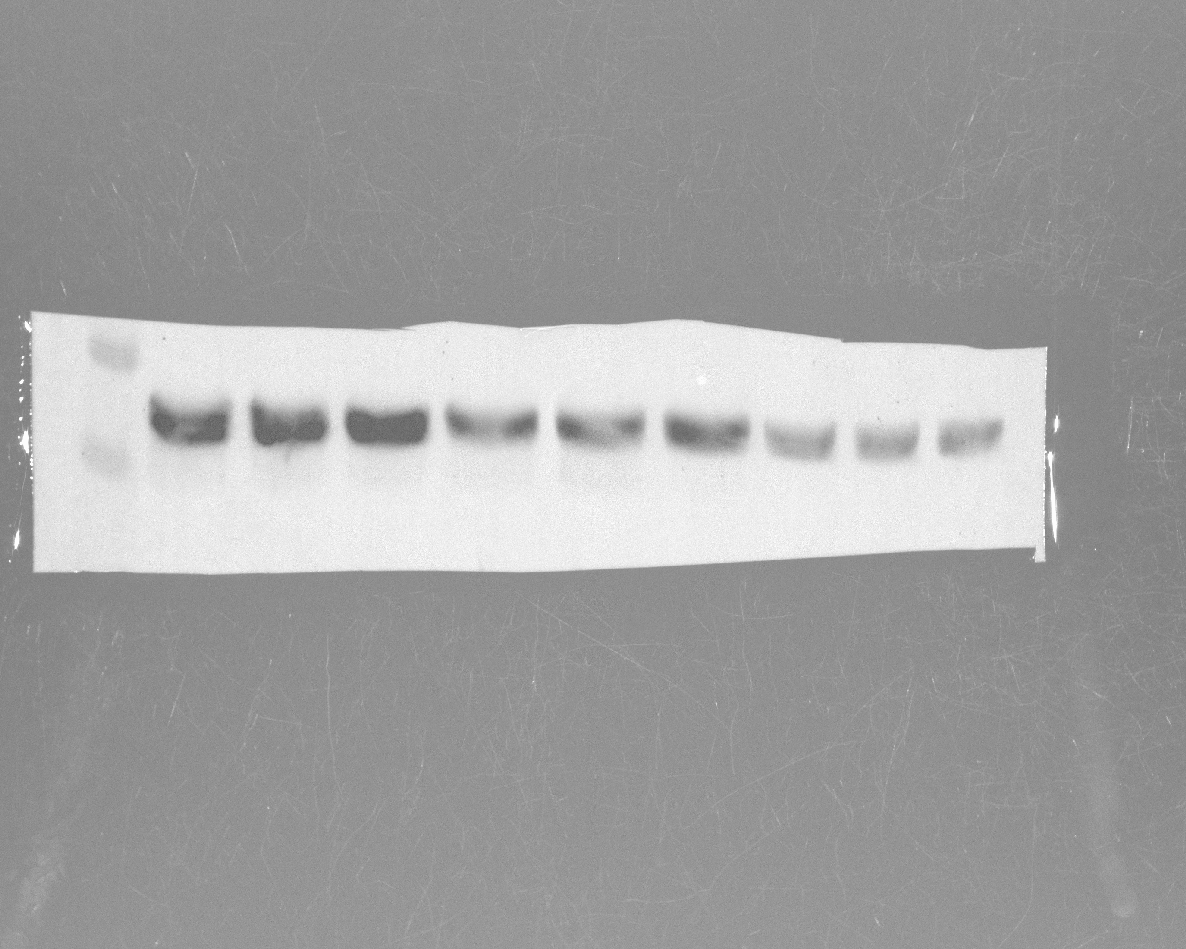

Supplement: Figure 6—source data 1. [file elife-108883-fig6-data1.zip › Figure 6-source data/Figure 6-source data 2.tif]

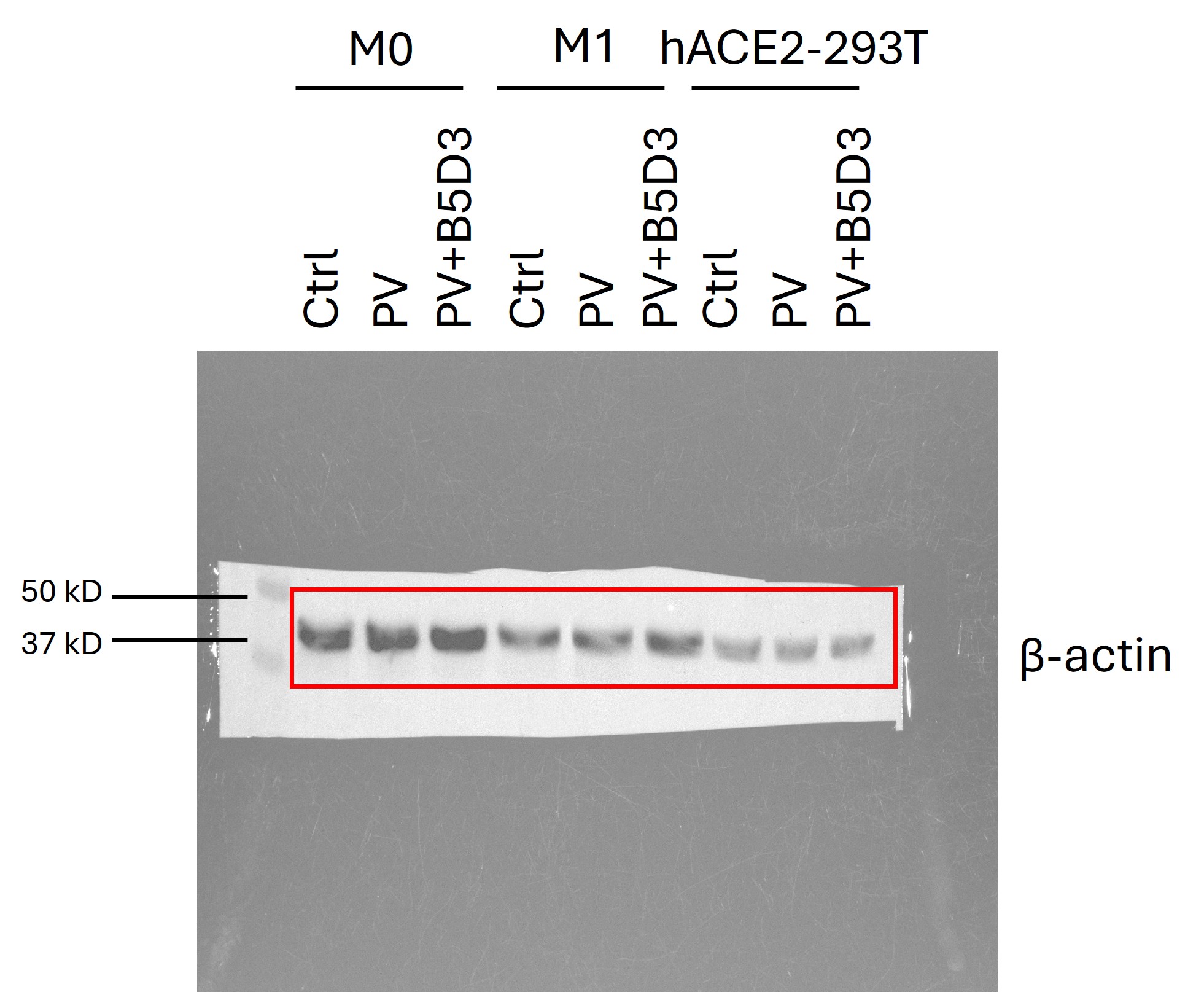

Supplement: Figure 6—source data 2. [file elife-108883-fig6-data2.zip › Figure 6_source data_labelled/Figure 6-source data 2_labelled.jpg]

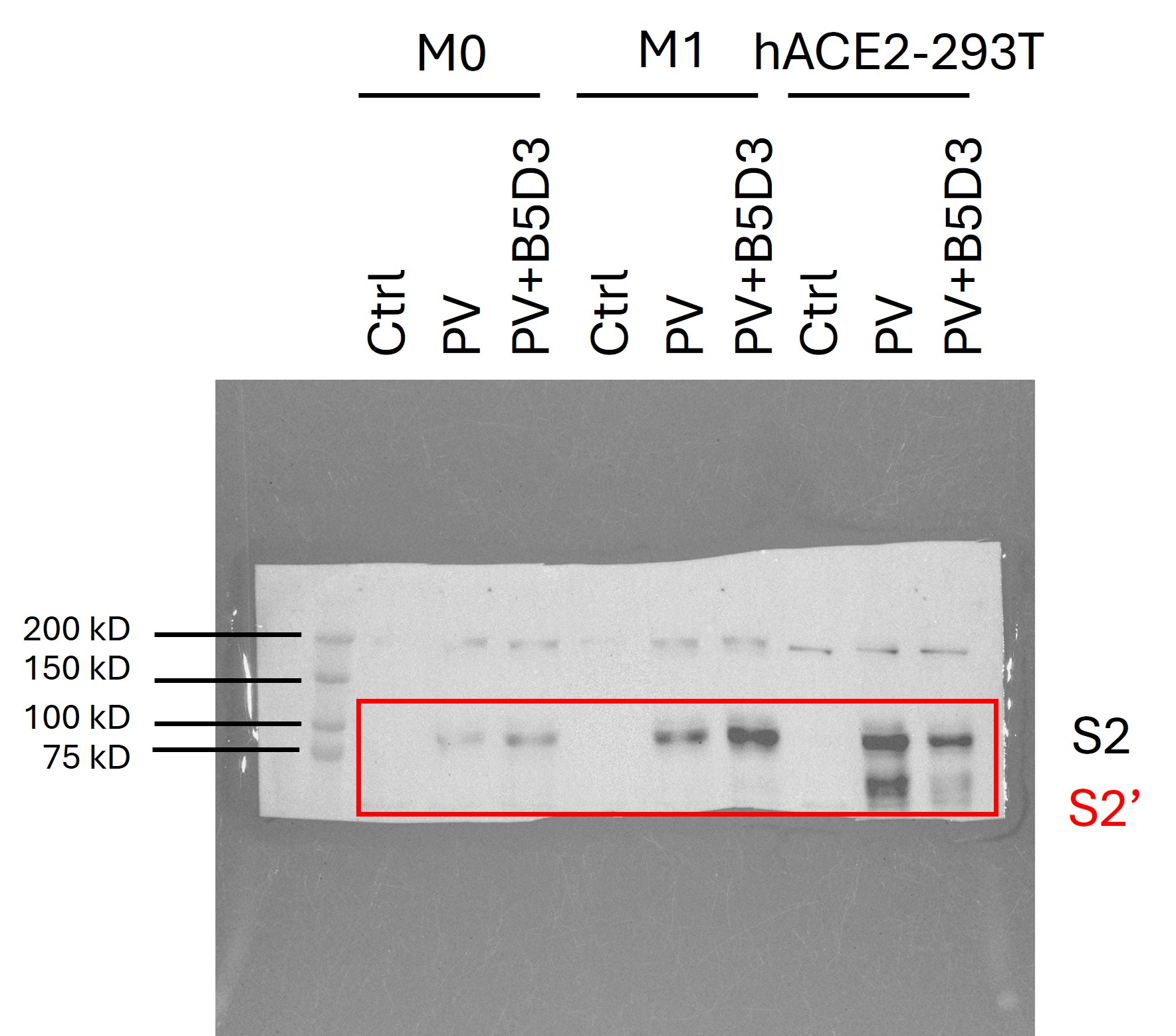

Supplement: Figure 6—source data 2. [file elife-108883-fig6-data2.zip › Figure 6_source data_labelled/Figure 6-source data 1_labelled.jpg]
