# Supplementary material for: Redirection of SARS-CoV-2 to phagocytes by intranasal sACE2-Fc as a universal decoy confers complete prophylactic protection
Source: eLife. 2026 May 18;14:RP108883. doi: 10.7554/eLife.108883 (PMC13183376; doi:10.7554/eLife.108883)
Supplement: Supplementary file 1. [file elife-108883-supp1.docx]

Supplementary File 1 for

**Redirection of SARS-CoV-2 to Phagocytes by Intranasal sACE2-Fc as a Universal Decoy Confers Complete Prophylactic Protection**

Jingyi Wang *et al.*

Corresponding authors: Leo L. Poon, llmpoon@hku.hk; Bo Feng, fengbo@cuhk.edu.hk

**Supplementary File 1A. Enhanced tolerance and stability of B5-derivatives compared to WT sACE2-Fc in AAV-administered K18-hACE2 mice.** Detailed *post hoc* comparisons among treatment groups shown in **Figure 1–figure supplement 4B**. Diff., difference; **, 0.001 ≤ *p* < 0.01; *, 0.01 ≤ *p* < 0.05. *p* values were determined by Tukey’s multiple comparisons test.

| Tukey's multiple comparisons test | Mean Diff. | Adjusted  *p* value | Summary |
| --- | --- | --- | --- |
| PBS vs. WT | -9990 | 0.0017 | ** |
| PBS vs. B5-D3 | -29104 | 0.0046 | ** |
| PBS vs. B5-D4 | -29894 | 0.0028 | ** |
| PBS vs. B5-D5 | -26774 | 0.0013 | ** |
| WT vs. B5-D3 | -19114 | 0.0145 | * |
| WT vs. B5-D4 | -19904 | 0.0078 | ** |
| WT vs. B5-D5 | -16784 | 0.0021 | ** |
| B5-D3 vs. B5-D4 | -790.4 | 0.9459 | ns |
| B5-D3 vs. B5-D5 | 2330 | 0.4915 | ns |
| B5-D4 vs. B5-D5 | 3120 | 0.2103 | ns |

**Supplementary File 1B. Primers used in quantitative PCR.**

| Primers | Sequences |
| --- | --- |
| SARS-CoV-2 *S* | CGTCAGGCTGTTTAATAGGGGC |
|  | GACTAGCTACACTACGTGCCCGC |
| SARS-CoV-2 *N* | CAATGGCGGTGATGCTGCTCTTG |
|  | TTCTGGACCACGTCTGCCGAAAG |
| AAV copies | AGCCGGAGAACAACTACAAG |
|  | CCAACACACAGATCTAATG |
| Mouse *Gapdh* | GGGCATCTTGGGCTACACTGAGGACCAG |
|  | CACCCTGTTGCTGTAGCCGTATTCATTGTC |
